# Trends of Non-Accidental, Cardiovascular, Stroke and Lung Cancer Mortality in Arkansas Are Associated with Ambient PM_2.5_ Reductions 

**DOI:** 10.3390/ijerph110707442

**Published:** 2014-07-21

**Authors:** Marie-Cecile G. Chalbot, Tamara A. Jones, Ilias G. Kavouras

**Affiliations:** Department of Environmental and Occupational Health, College of Public Health, University of Arkansas for Medical Sciences, Little Rock, AR 72205, USA; E-Mails: mchalbot@uams.edu (M.-C.G.C.); misstiat94@yahoo.com (T.A.J.)

**Keywords:** air pollution, fine particles, annual trends, spatial variation, urban aerosol

## Abstract

The cardiovascular and stroke mortality rates in Arkansas are among the highest in the USA. The annual trends of stroke and cardiovascular mortality are barely correlated to smoking cessation; while the prevalence of risk factors such as obesity; cholesterol and hypertension increased over the 1979–2007 period. The study determined the effect of chronic exposure to PM_2.5_ on non-accidental; cardiovascular; stroke and lung cancer mortality in Arkansas over the 2000–2010 period using the World Health Organization’s log-linear health impact model. County chronic exposures to PM_2.5_ were computed by averaging spatially-resolved gridded concentrations using PM_2.5_ observations. A spatial uniformity was observed for PM_2.5_ mass levels indicating that chronic exposures were comparable throughout the state. The reduction of PM_2.5_ mass levels by 3.0 μg/m^3^ between 2000 and 2010 explained a significant fraction of the declining mortality. The effect was more pronounced in southern and eastern rural Arkansas as compared to the rest of the state. This study provides evidence that the implementation of air pollution regulations has measurable effects on mortality even in regions with high prevalence of major risk factors such as obesity and smoking. These outcomes are noteworthy as efforts to modify the major risk factors require longer realization times.

## 1. Introduction

The southeast region of the USA has been historically identified as the “stroke belt” but more recently, the existence of a “heart failure belt” was also observed in the same region [1,2]. Arkansas has the highest age-adjusted stroke mortality in the USA and ranked among the top five states for heart disease mortality. The 2005–2009 age-adjusted mortality rates for heart disease and stroke was higher among African-Americans than for whites and Hispanics [3]. Heart disease and stroke mortality rates were higher in rural communities in eastern Arkansas along the Mississippi Delta region as compared to the urbanized areas in central and northwest Arkansas. Deaths from heart diseases and stroke have been decreasing over the past 20 years [3]; however, the prevalence of major risk factors such as obesity, high blood pressure and cholesterol increased [4]. Changes in smoking habits were associated with long-term trends of mortality rates but did not justify the observed racial and geographical disparities in southeast USA [4,5]. The anticipated improvements of increased awareness, new drugs and therapies did not materialize shortly after their implementation [4].

Owing to the absence of major industrial facilities, scarcely populated urban areas and frequent precipitation scavenging, the impacts of atmospheric particulate pollution on cardiovascular and stroke mortality received very little attention, if any, in this region. Meanwhile, epidemiological studies showed consistent, strong and statistically significant associations between PM_2.5_ (fine particles with aerodynamic diameter less than 2.5 μm) and cardiovascular, stroke and respiratory mortality and morbidity in the USA and around the world [6,7,8,9,10,11,12]. Recent studies showed that the risk of stroke may increase up to 34% even at low PM_2.5_ levels in New England [13]. These levels were comparable or lower to those typically measured in Arkansas [14]. Secondary sulfate and nitrate, biomass combustion, traffic and diesel particles were identified as the most important particle types and sources of PM_2.5_ in Arkansas, which is typical to those observed in eastern USA [14,15,16]. Emissions of pollutants from energy-related sources (*i.e.*, volatile organic compounds and nitrogen oxides) from upwind regions (oil/natural gas exploration and refineries in the Gulf of Mexico, Houston, TX, USA) were significant determinants of PM_2.5_ levels in Arkansas. Previous studies showed that transport of volatile organic compounds and nitrogen oxides from southeast USA along the Gulf Coast yielded very high ozone (O_3_) concentration along the Mississippi Valley to the Great Lakes and Upper Midwest region [17]. 

The overarching aim of this study was to determine the burden of chronic exposures to PM_2.5_ on non-accidental, cardiovascular, stroke and lung cancer mortality in the state of Arkansas over the 2000–2010 period. We achieved this by using the World Health Organization’s (WHO) health impacts analysis (HIA) methodology [18,19,20,21,22]. Estimates of the PM_2.5_ concentrations, the annual trends, spatiotemporal variability and county-average PM_2.5_ mass concentrations were computed using PM_2.5_ measurements in multiple locations. 

## 2. Methods

### 2.1. Health Impacts Assessment

We obtained county-based data for non-accidental, cardiovascular (ICD-10 codes: 410–429), stroke (ICD-10 codes: 430–438) and lung cancer (ICD-10 code: 162) mortality from the Arkansas’s Department of Health Indicator Based Information System for Public Health. The 2000 and 2010 population counts of 30+ years old for each county were obtained from USA Census. 

The effects of changes in PM_2.5_ between 2000 and 2010 were assessed by calculating the change in the number of deaths (Y, in Equation (1)) in 2010 for each county where P is the 2010 population in each county, b is the 2000 mortality rate (per 100,000), β is the effect coefficient per a 1 μg/m^3^ increase of PM_2.5_ mass, and Δ(PM_2.5_) is the change in PM_2.5_ mass between 2001 and 2010 [18,19,20]:

Y = (P/100,000) × b × (e^β·Δ(PM_2.5_)^ - 1) (1)

The β values for non-accidental (1.06; 95% CI: 1.02–1.11), lung cancer (1.14; 95% CI: 1.04–1.23) and cardiovascular (1.09; 95% CI: 1.03–1.06) mortality were obtained from the American Cancer Society (ACS) study, a long-term cohort study examining the impacts of air pollution on mortality [23,24,25]. The relative risk of PM_2.5_-induced stroke mortality (1.03; 95% CI: 0.02–2.04) were obtained from the analysis of the associations between PM_2.5_ and mortality in 27 USA communities including Birmingham, Alabama and Memphis, Tennessee, two urban areas with similar demographic characteristics to Arkansas urban areas [26]. 

The PM_2.5_-resultant change in mortality between 2000 and 2010 (Y in Equation (1)) was then compared to the actual change in mortality between 2000 and 2010 and the percentage effect of PM_2.5_-resultant mortality on observed mortality (ΔΜ) was calculated as follows:

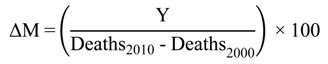
(2)
where Deaths_2000_ and Deaths_2010 _were the number of non-accidental, cardiovascular, stroke and lung cancer deaths in 2000 and 2010, respectively. Because of the observed declining trend of PM_2.5_, a reduction in mortality was anticipated. Thus, positive ΔΜ values indicated the magnitude of the reduction of PM_2.5_-resultant deaths to the overall reduction of deaths, while, negative values showed that the actual number of deaths increased between 2000 and 2010. ΔΜ > 1 (or 100%) suggested that the actual reduction in deaths was lower than the estimated reduction due to PM_2.5_ declining levels.

### 2.2. PM_2.5_ Mass Concentrations

**Figure 1:**
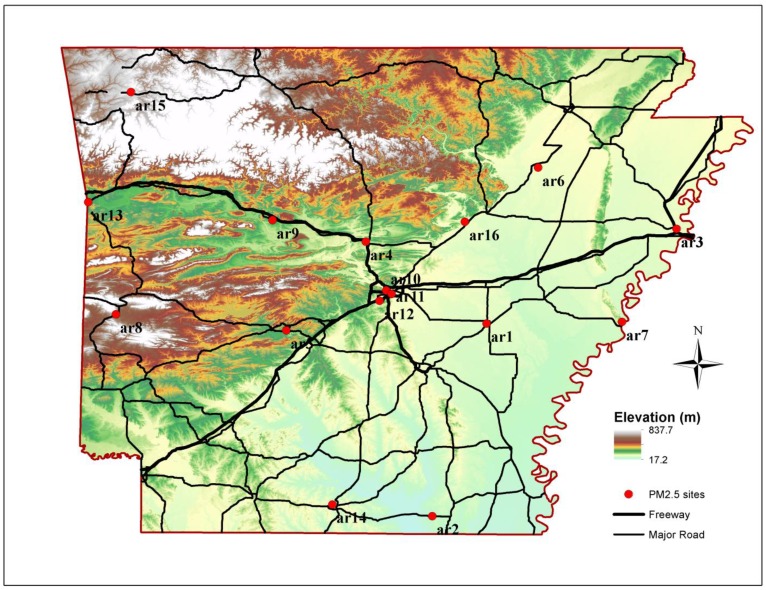
The locations of the PM_2.5_ monitoring sites in Arkansas.

The measured PM_2.5_ mass concentrations in eighteen (18) sites in the state of Arkansas during the 2000–2011 timespan were retrieved from EPA’s Air Quality System (AQS). Figure 1 shows the locations of the monitoring sites in the state including those in the Little Rock/North Little Rock metropolitan statistical area (ar11, ar11 and ar12 in Figure 1). Sixteen sites including the NCore site (ar10; AQS #05-119-0007; a site that integrates advanced measurement for particles, pollutant gases and meteorology), are operated by the Arkansas Department of Environmental Quality. Monthly mean PM_2.5_ concentrations were computed for periods with more than 75% valid measurements (22 days/month for daily measurements and 7 days/month for 1-in-3 measurement frequency). The monthly 24-h mean PM_2.5_ mass concentration was computed for months with more than 75 percent valid estimates of daily PM_2.5_ mass concentrations. The annual trend of PM_2.5_ mass in each site was computed by ordinary least squares regression analysis of deseasonalized monthly mean PM_2.5_ mass concentrations using the “Census I” method integrated in SPSS [14,27]. Missing monthly averages was replaced by average concentration of the same month over the 2000–2010 period introducing an error of up to 1.6% for 10% missing values (no more than 10% of missing values were observed in our dataset).

The absolute (ΔC) and the relative (%ΔC/Ref) differences of daily PM_2.5_ mass concentrations between two sites were computed to evaluate concentration gradients in the region [28,29]. The PM_2.5_ monitor at the NCore site was the reference site because was centrally located to the study region, had the most complete PM_2.5_ dataset and detailed data of PM_2.5_ chemical content and other criteria air pollutants were also available. The relative concentration differences were computed as the percentage of the absolute concentration difference to the reference site concentration. Positive values indicate that PM_2.5_ concentrations at the site were higher than those measured at the NCore site. The coefficient of divergence (COD) was used to assess the spatial uniformity of measurements with respect to the concentration levels (Equation (3)) [28,29].


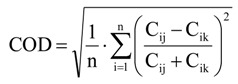
(3)
where *n* is the total number of paired measurements, and C*_ij_* and C*_ik_* are the measured concentrations at the reference and comparison sites on the *i*-th month, respectively. COD values vary from 0 to 1, with COD values close to unity being suggestive of strong spatial variation [28,29].

The mean annual PM_2.5_ mass concentration for each county was computed by calculating the average of annual PM_2.5 _mass concentration for 0.2° × 0.2° using kriging and accounting for elevation boundaries and restrictions in ArcMAP (Version 10.0, ESRI, Redlands, CA, USA) [30,31,32]. 

## 3. Results

### 3.1. PM_2.5_ Annual and Spatial Trends

Table 1 presents the mean and standard deviation (σ) of the PM_2.5_ mass concentrations during the study period, the annual PM_2.5_ trend (±standard error (s)), the median and σ of absolute and relative concentration differences and the mean (±s) COD value for each of the 18 sites. The mean PM_2.5_ mass levels demonstrated very little spatial variability, from 11.0 to 13.2 μg/m^3^. The highest mean PM_2.5_ mass was measured in the Little Rock/North Little Rock sites (ar10, ar11 and ar12) and nearby populated regions, from 12.0 to 13.2 μg/m^3^ (ar4 site in Conway and ar9 site in Russellville) followed by PM_2.5_ levels measured in the West Memphis area (ar3). The lowest PM_2.5_ levels in populated areas were measured in the southwest (ar8) and northwest (ar15) regions. 

**Table 1:** The PM_2.5_ annual trends (mean ± st.error), absolute (ΔC) and relative (%ΔC/Ref) concentration differences (compared to the “ar10” site) and the COD ratio (mean ± standard error) in Arkansas monitoring sites.

Site	Annual Trend (μg/m^3^/year)	ΔC		%ΔC/Ref		COD
		*Median*	*SD*	*Median*	*SD*	
**ar1**	−0.3 ± 0.5	−0.9	3.5	−8.1	74.5	0.19 ± 0.02
**ar1**	−0.5 ± 0.6	−1.0	4.8	−8.2	122.8	0.19 ± 0.03
**ar3**	−0.4 ± 0.5	−0.5	4.4	−4.4	83.5	0.18 ± 0.01
**ar4**	−0.3 ± 0.5	−0.7	3.1	−6.2	103.5	0.19 ± 0.03
**ar5**	−0.2 ± 0.5	−1.0	3.4	−9.3	31.7	0.20 ± 0.04
**ar6**	−0.2 ± 1.0	−1.1	4.5	−10.3	120.3	0.22 ± 0.05
**ar7**	−0.3 ± 0.6	−1.1	4.5	−9.1	87.8	0.20 ± 0.04
**ar8**	−0.1 ± 0.3	−1.5	4.4	−13.4	109.9	0.21 ± 0.06
**ar9**	−0.2 ± 0.4	−0.7	4.0	−5.9	36.3	0.18 ± 0.01
**ar10**	−0.3 ± 0.4	-	-	-	-	-
**ar11**	−0.3 ± 0.5	0.3	3.0	2.5	88.7	0.16 ± 0.01
**ar12**	−0.3 ± 0.5	0.1	2.7	1.3	58.3	0.16 ± 0.01
**ar13**	−0.1 ± 0.5	−0.7	4.4	−6.9	127.5	0.19 ± 0.03
**ar14**	−0.3 ± 0.5	−0.6	4.6	−5.7	134.0	0.18 ± 0.04
**ar15**	−0.2 ± 0.3	−1.4	4.7	−13.9	39.3	0.21 ± 0.05
**ar16**	−0.3 ± 0.5	−0.7	3.8	−6.1	108.3	0.20 ± 0.04

The highest absolute (ΔC) and relative (ΔC/Ref) concentration differences varied from −1.1 (for ar6 and ar7 sites) to 0.3 μg/m^3^ (for ar11) and from −13.9% to 2.5%, respectively. The COD values for each site range from 0.16 ± 0.00 to 0.22 ± 0.05. For the two background sites, absolute and relative concentration differences were higher than those computed for the urban areas. PM_2.5_ decreased from 0.5 ± 0.6 to 0.1 ± 0.3 μg/m^3^ per year with the highest declines in southern and eastern Arkansas (ar1, ar2, ar3 and ar7, Figure 1).

### 3.2. Mortality Reductions

**Figure 2:**
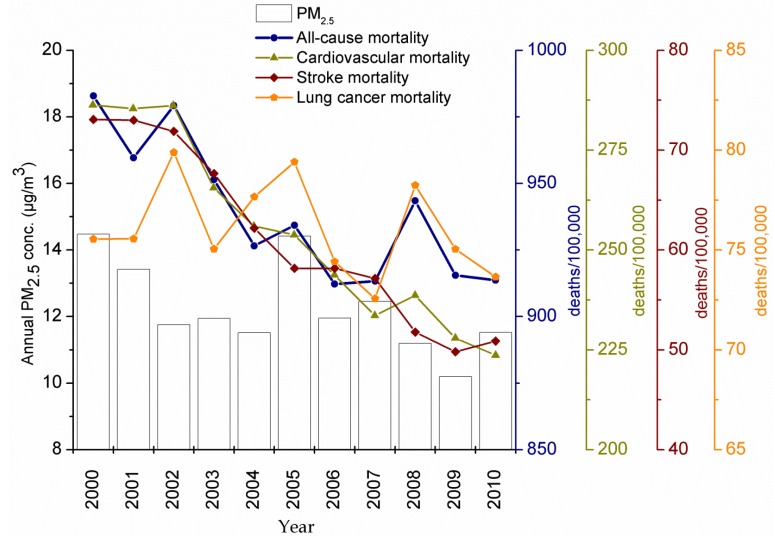
Annual trends of PM_2.5_ mass concentration, and non-accidental, cardiovascular, stroke and lung cancer mortality in Arkansas.

Figure 2 shows the annual trends of mortality rates for non-accidental, cardiovascular, stroke and lung cancer mortality and the mean PM_2.5_ mass concentration in Arkansas. For all variables, a decreasing trend was observed, which was more pronounced for cardiovascular (about 21%) and stroke mortality (~30%). Non-accidental mortality decreased by 7% between 2000 and 2010 with significant inter-annual variability that resembled that of lung cancer. For lung cancer, the mortality declined by only 2%. The annual PM_2.5_ mass concentration in Arkansas decreased from 14.5 μg/m^3^ in 2000 to 11.5 μg/m^3^ in 2010 (~21%). 

**Figure 3:**
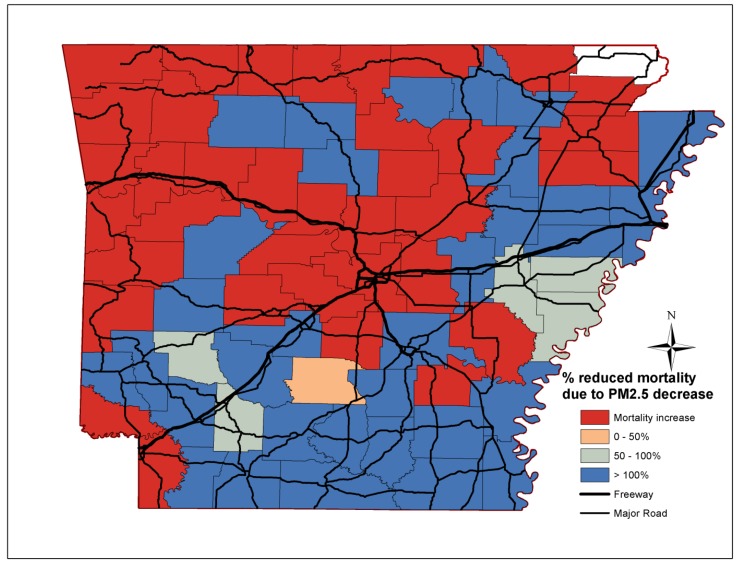
Percentage reduction of non-accidental mortality attributed to PM_2.5_ reductions for each Arkansas county.

**Figure 4:**
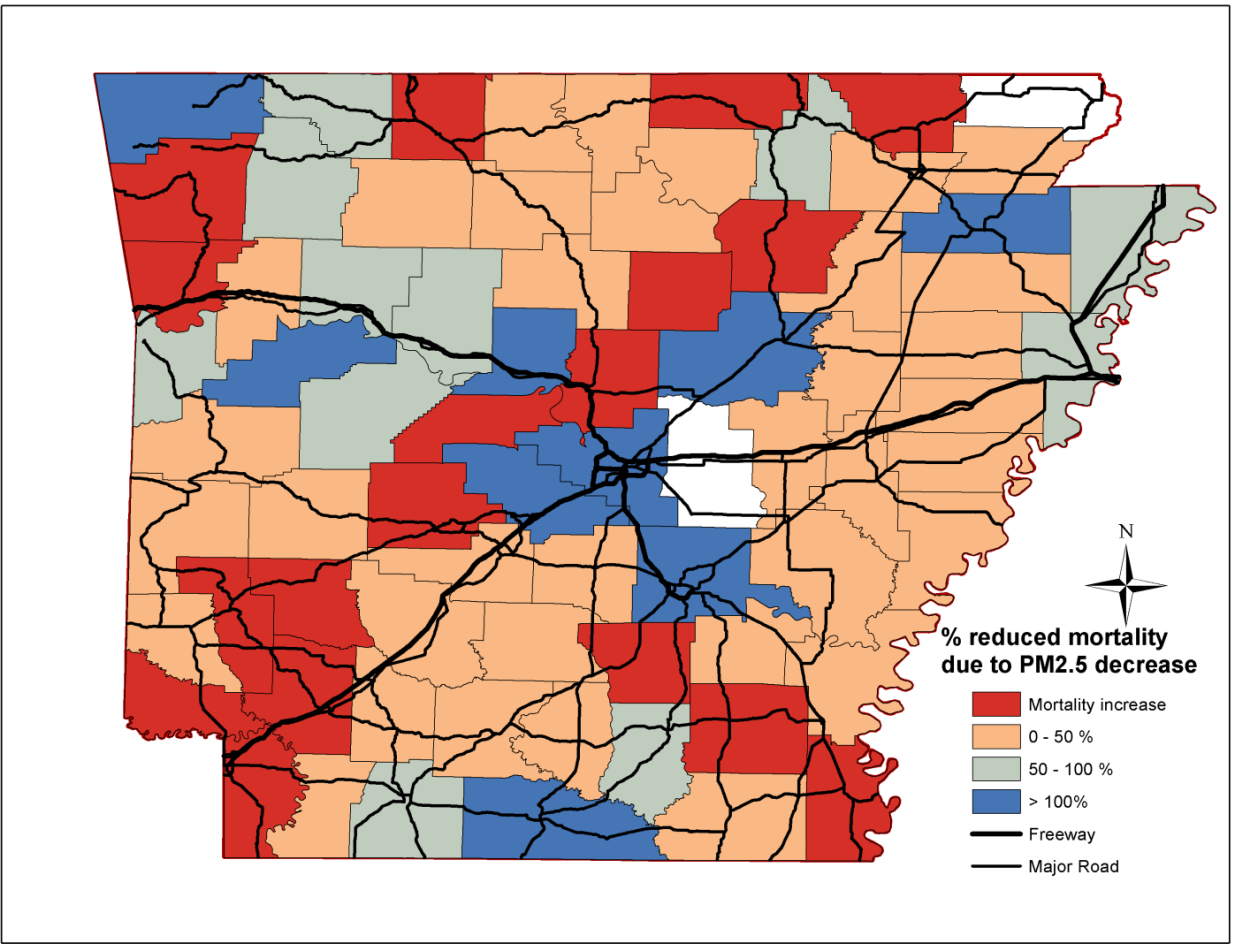
Percentage reduction of cardiovascular mortality attributed to PM_2.5_ reductions for each Arkansas county.

**Figure 5:**
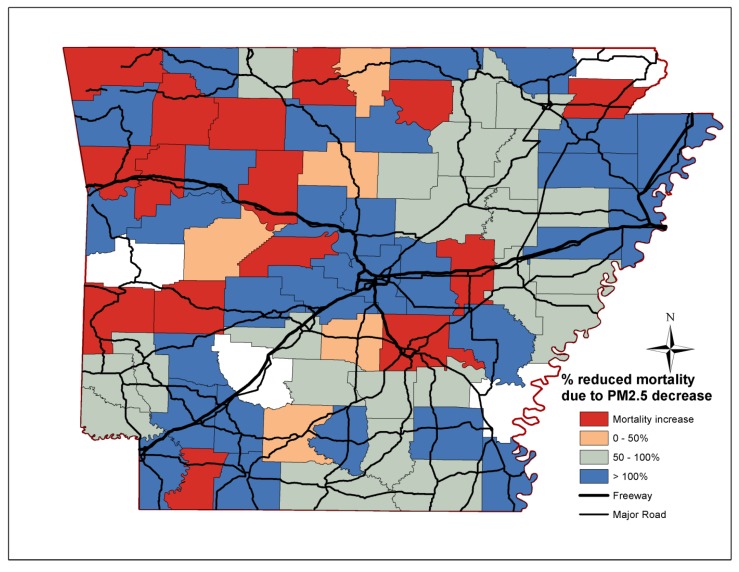
Percentage reduction of stroke mortality attributed to PM_2.5_ reductions for each Arkansas county.

**Figure 6:**
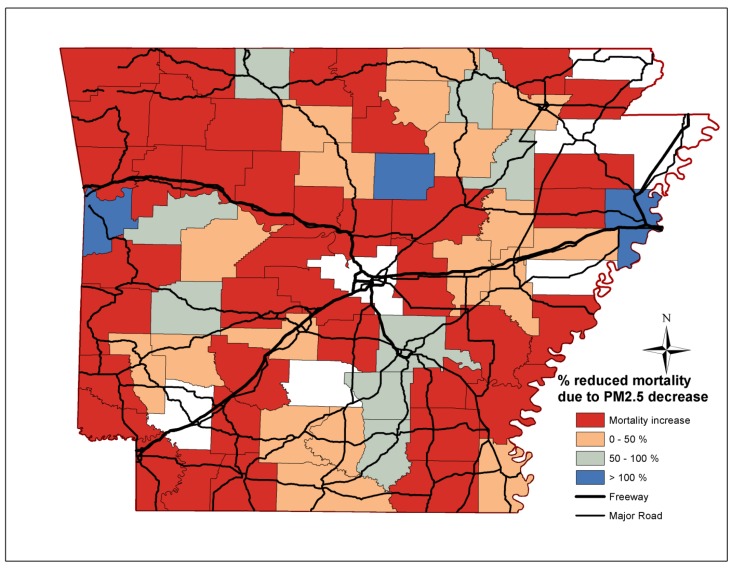
Percentage reduction of lung cancer mortality attributed to PM_2.5_ reductions for each Arkansas county.

The percentage reductions of non-accidental, cardiovascular, stroke and lung cancer mortality attributed to reductions in PM_2.5_ mass concentrations per county in Arkansas are depicted in Figure 3, Figure 4, Figure 5 and Figure 6 respectively. Out of the 75 counties, cardiovascular, stroke and lung cancer mortality increased in 13, 15 and 40 counties from 2000 to 2010, respectively, mostly in western and central Arkansas. For cardiovascular mortality, the reduction of PM_2.5_ in the Little Rock/North Little Rock, Jonesboro and Bentonville area fully accounted for the declining mortality rates. For rural Arkansas and the Delta region, up to 50% of reduced premature cardiovascular deaths was due to PM_2.5_ reductions. 

The improvements in PM_2.5_ mass concentrations prevented the vast majority of premature deaths due to stroke throughout Arkansas, especially in the farming communities along the Delta region (eastern/southeast Arkansas). Less than 50% of the prevented lung cancer deaths were due to lower PM_2.5_ levels. The decline in non-accidental, cardiovascular and stroke mortality was due to the reduced and less-severe incidences, while, the efficacy of lung cancer treatment did not improve over the last decades [33]. 

## 4. Discussion

In this study, we demonstrated the absence of a strong PM_2.5_ spatial concentration gradient. Slightly higher PM_2.5_ mass concentrations were measured at populated areas (*i.e.*, Little Rock, Conway, West Memphis and Russellville) due to the increased impacts from traffic and other anthropogenic sources typically present in urban centers. Low ΔC, ΔC/Ref and COD values were previously observed for urban areas that are highly impacted by secondary PM_2.5_ and for ozone, a pollutant that is formed from the oxidation of nitrogen oxides and volatile organic compounds during transport [28,34]. In these cases, the spatial uniformity was attributed to long residence times allowing for dispersion and diffusion [28]. In a previous study, we identified that a large fraction of PM_2.5_ originated from sources outside the state of Arkansas [14]. During winter, ammonium nitrate particles formed during transport of nitrogen oxides from anthropogenic activities in western USA and ammonia from livestock and agriculture in Midwest were observed. On the other hand, sulfate (from SO_2_ oxidation) and diesel combustion particles from power plants, oil refineries, shipping and oil/natural gas exploration facilities from Gulf Mexico coast and Texas dominated PM_2.5_ in the summer. These types of secondary aerosol ensure a certain degree of uniformity [28]. While the kriging method lacks the ability to identify short-term hot-spots (*i.e.*, areas with episodic high PM_2.5_ mass concentrations), alternative approaches such as PM_2.5_ mass concentration at the nearest monitoring site fail to spatially describe PM_2.5_ mass concentrations. The use of satellite aerosol optical depth was also suggested; however, the coverage and reliability of these measurements were extremely poor before 2007 [35,36].

The annual PM_2.5_ mass levels mildly decreased and remained slightly below the annual ambient air quality national standard (NAAQS) of 15 μg/m^3^ over the 2000–2010 period. The decline was higher for the southern-southeast part of the state and along the Mississippi river valley, a region that was influenced by transport of sulfate and diesel particles emitted from the Gulf of Mexico off-shore and coastal sources. We previously showed that 75% of PM_2.5_ reductions were due to sulfate and diesel particles and it was attributed to significant controls of SO_2_ emissions from coal-fired power plants [14]. On the other hand, reduction of nitrate particles affecting mostly northwest and central Arkansas was less abundant because of the limited controls on ammonia emissions from livestock and agriculture. These results suggested that while there was no significant difference in PM_2.5_ mass levels across the state of Arkansas, particle types and chemical content in southern/southwest Arkansas may be different than those in central and northwest Arkansas. These trends were also consistent with the spatial and temporal variation of PM_2.5_ chemical species across the USA [15].

We also demonstrate that the decrease in non-accidental, cardiovascular, stroke and to a lesser extent for lung cancer deaths may be partially attributed to the reduction in annual PM_2.5_ mass concentrations. The decline in cardiovascular and stroke mortality coincided with efforts to control cardiovascular risk factors; however, the effect of specific actions on reduced mortality rates cannot be assessed [37]. The effect of PM_2.5_ on mortality was more pronounced in the southern and eastern counties than central and northwest counties in Arkansas. In many cases, the benefit of the reduced PM_2.5_ mass levels exceeded the decrease in deaths suggesting that other risk factors were also declining. On the contrary, the prevalence of a risk factor (*i.e.*, hypertension, smoking or nutrition) was among the highest in the state for counties in which mortality increased. Epidemiological studies consistently determine statistically significant associations between ambient PM_2.5_ and respiratory and cardiovascular mortality and morbidity, but more importantly, they report different effects for particle types and chemical species. In a recent analysis of mortality and PM_2.5_ trends in 25 communities from 2000 to 2005, Franklin *et al.* [38] determined that sulfate (0.51% increase), potassium (0.41% increase), silicon (0.41% increase), aluminium (0.58% increase), bromine (0.38% increase), nickel (0.37% increase), vanadium (0.28% increase) were major modifiers of the PM_2.5_-mortality associations. Ni, V, Br are tracers of oil and gas combustion, while Al and Si may be associated with industrial activities and minor quantities of mineral soil particles. For comparison, the effect modifications of nitrate, ammonium and organic particles were 0.04%, −0.49% and −0.02%. Stronger associations were observed in east USA during spring and summer where sulfate is the largest contributor to PM_2.5_ than in the west USA. Similarly, the comparison of mortality rates in Seattle and Detroit revealed the important effect of secondary and oil combustion particles [12].

There are several limitations for this assessment. The first one is related to the accuracy of exposures to ambient PM_2.5_ mass concentrations using county-based averages of gridded PM_2.5_ estimates based on kriging interpolation of PM_2.5_ measurements. This approach would be associated with large uncertainties of estimates of daily PM_2.5 _levels but they are reduced as the averaging period is increased to annual estimates [30]. The use of annual levels was also a better determinant of the effects of chronic PM_2.5_ exposures to mortality [19]. The second limitation is associated with the representativeness of relative risk estimates for populations with different ethnic, racial and socioeconomic characteristics since most of the epidemiological studies have been done in densely populated urban areas. African-Americans have higher risks of heart disease and stroke than people of other races [3]. The risk of Hispanic and Native Americans for heart disease is also higher than that of Caucasian origin. In addition, exposures to PM_2.5_ chemical components differed by age, race/ethnicity and socioeconomic status [39]. Here, we used relative risk estimates obtained from large studies that included subjects with demographic characteristics comparable to those in Arkansas and communities near Arkansas (*i.e.*, Memphis, TN and Birmingham, AL, USA). 

The findings of this study are also relevant for policy-relevant matters such as they provide strong indications on the benefits of the Clean Air Act and the National Ambient Air Quality Standards (NAAQS) legislations implementation. They also highlight the need to design targeted policies to reduce the burden of the most harmful types of particles on health taking into account regional characteristics. For example, controls on emissions of ammonia and nitrogen oxides would reduce the formation of nitrate particles in winter but have very little effect during summer, because low ambient temperatures are needed for the gas-to-particle partitioning of nitric acid. These borderline but real effects of PM_2.5_ on reduced mortality are also necessary to provide sufficient lead time for policies and efforts targeting major cardiovascular and stroke risk factors (obesity, hypertension, lack of exercise, cholesterol, smoking and health care access) to emerge. For example, meaningful improvements of the obesity rate may be observed between generations rather than within a generation. Similarly, reduction in smoking is typically associated with reduced smoking frequency rather than complete cessation. Advances in medicine and health technologies could only help those who have access to health care, thus reducing their overall effectiveness. It is, therefore, essential to keep making incremental changes in mortality rates by reducing the burden of air pollution and maintain the involvement of policy makers and the public.
